# Wide variation in sexually transmitted infection testing and counselling at Aboriginal primary health care centres in Australia: analysis of longitudinal continuous quality improvement data

**DOI:** 10.1186/s12879-017-2241-z

**Published:** 2017-02-15

**Authors:** Barbara Nattabi, Veronica Matthews, Jodie Bailie, Alice Rumbold, David Scrimgeour, Gill Schierhout, James Ward, Rebecca Guy, John Kaldor, Sandra C. Thompson, Ross Bailie

**Affiliations:** 1 Western Australian Centre for Rural Health, University of Western Australia, 167 Fitzgerald Street, Geraldton, WA 6530 Australia; 2 Menzies School of Health Research, Brisbane, QLD Australia; 3 University Centre for Rural Health, University of Sydney, Lismore, NSW Australia; 4 The Robinson Research Institute, The University of Adelaide, Adelaide, Australia; 5 Spinifex Health Service Tjuntjuntjara, Kalgoorlie, WA Australia; 6 The Kirby Institute, University of New South Wales, Sydney, NSW Australia; 7 South Australian Health and Medical Research Institute, Adelaide, South Australia

**Keywords:** Aboriginal and Torres Strait Islander populations, Sexually transmitted infections, Primary health care, Quality improvement, Clinical audits, Variation in care, Indigenous

## Abstract

**Background:**

Chlamydia, gonorrhoea and syphilis are readily treatable sexually transmitted infections (STIs) which continue to occur at high rates in Australia, particularly among Aboriginal Australians. This study aimed to: explore the extent of variation in delivery of recommended STI screening investigations and counselling within Aboriginal primary health care (PHC) centres; identify the factors associated with variation in screening practices; and determine if provision of STI testing and counselling increased with participation in continuous quality improvement (CQI).

**Methods:**

Preventive health audits (*n* = 16,086) were conducted at 137 Aboriginal PHC centres participating in the Audit and Best Practice for Chronic Disease Program, 2005–2014. STI testing and counselling data were analysed to determine levels of variation in chlamydia, syphilis and gonorrhoea testing and sexual health discussions. Multilevel logistic regression was used to determine factors associated with higher levels of STI-related service delivery and to quantify variation attributable to health centre and client characteristics.

**Results:**

Significant variation in STI testing and counselling exists among Aboriginal PHC centres with health centre factors accounting for 43% of variation between health centres and jurisdictions. Health centre factors independently associated with higher levels of STI testing and counselling included provision of an adult health check (odds ratio (OR) 3.40; 95% Confidence Interval (CI) 3.07-3.77) and having conducted 1–2 cycles of CQI (OR 1.34; 95% CI 1.16-1.55). Client factors associated with higher levels of STI testing and counselling were being female (OR 1.45; 95% CI 1.33-1.57), Aboriginal (OR 1.46; 95% CI 1.15-1.84) and aged 20–24 years (OR 3.84; 95% CI 3.07-4.80). For females, having a Pap smear test was also associated with STI testing and counselling (OR 4.39; 95% CI 3.84-5.03). There was no clear association between CQI experience beyond two CQI cycles and higher levels of documented delivery of STI testing and counselling services.

**Conclusions:**

A number of Aboriginal PHC centres are achieving high rates of STI testing and counselling, while a significant number are not. STI-related service delivery could be substantially improved through focussed efforts to support health centres with relatively lower documented evidence of adherence to best practice guidelines.

## Background

Preventable and curable sexually transmitted infections (STIs) including chlamydia, gonorrhoea and syphilis continue to be a major cause for concern in Australia, particularly among Aboriginal and Torres Strait Islander (hereafter respectfully referred to as Aboriginal) Australians. There are large disparities in STI notification rates between Aboriginal and non-Aboriginal Australians with gonorrhoea, syphilis, and chlamydia, 10 times, 6 times and 3 times higher among Aboriginal compared to non-Aboriginal Australians [1]. Among Aboriginal Australians, young people aged 15–29 years are the most affected group, accounting for 82% of chlamydia notifications and 72% of gonorrhoea notifications [2]. Furthermore, Aboriginal Australians living in remote and very remote areas are burdened by significantly higher rates of STIs compared to those in urban and regional areas. For example, in 2015, the chlamydia notification rates among the Aboriginal population compared to the non-Aboriginal population was 2 times higher in major cities, 2 times higher in inner regional centres, 5 times higher in outer regional areas, 8 times higher in remote areas and 6 times higher in very remote areas. Disparities are even more marked for gonorrhoea, with notification rates 2 times higher in Aboriginal people compared to non-Aboriginal Australians in major cities and inner regional areas, 20 times higher in outer regional areas, 72 times higher in remote areas and 32 times higher in very remote areas [2]. For syphilis, rates in remote and very remote areas are 132 times and 70 times higher among Aboriginal Australians compared to non-Aboriginal Australians, respectively.

A number of guidelines provide recommendations on frequency of STI testing among Aboriginal clients attending primary health care (PHC) centres in Australia [3, 4], with National Aboriginal Community Controlled Health Organisation (NACCHO) preventive health guidelines recommending annual testing for chlamydia (15–29 year age group) and gonorrhoea (15–39 year age group) (see Table 1). Despite the high rates of STIs and efforts related to policy and practice, low rates of STI testing [5] and retesting [6] among eligible males and females in Australia continue to be documented. Over the past decade there have been a number of interventions to improve the quality of sexual health care for Aboriginal clients attending general practice in Australia including the Sexual Health Quality Improvement Project (SHIMMER) project in New South Wales (NSW) [7]; the STI in Remote communities: Improved and enhanced primary health care (STRIVE) project in the Northern Territory (NT), Queensland (QLD) and Western Australia (WA) [8]; and the Audit and Best Practice for Chronic Disease (ABCD) Program in the NT, QLD, NSW, WA and South Australia (SA) [9, 10]. The ABCD Program, in conjunction with One21seventy, a service support agency, supports systematic continuous quality improvement (CQI) in a wide range of community- and government-controlled PHC centres, providing opportunities for health centres to identify gaps and address barriers to evidence-based practice.

**Table 1:** Recommended schedule for testing for chlamydia, gonorrhoea, syphilis and adult health checks in Australia

Guideline	Test/STI	Target age group	Frequency
RACGP [4]	Chlamydia	Sexually active 15–29 years	Annually^a^
NACCHO/RACGP [3]	Chlamydia	- Sexually active 15–29 years	Annually
		- 30 years and above who are deemed to be at risk	
NACCHO/RACGP [3]	Gonorrhoea	Sexually active people aged 15–39 years	Annually
NACCHO/RACGP [3]	Sexual health counseling	All sexually active persons	Opportunistically
CARPA [43]	Adult health checks including screening for chlamydia, gonorrhoea and syphillis	Aboriginal people aged 15–54 years	2 yearly

^a^Repeat testing at 3–12 months if found to be positive and treated for chlamydia; CARPA Central Australian Rural Practitioners Association; NACCHO National Aboriginal Community Controlled Health Organisation; RACGP Royal Australian College of General Practitioners

Under the ABCD Program, health centres undertake preventive health clinical audits to assess documented provision of preventive health care for their clients, including delivery of sexual health care services, to determine levels of adherence to best practice. The audit processes allow health centres to benchmark themselves against other health centres, make judgements about their delivery of care, and determine priorities for improvement. Over 270 health centres have been involved in the ABCD/One21seventy program and 175 health centres voluntarily provided their audit data to the ABCD National Research Partnership (NRP) for further data analysis [11].

The aim of this study was to quantify the extent of variation in specific processes of care related to sexual health among Aboriginal primary health care centres using data obtained through the ABCD NRP. Specifically, the study aimed to: determine the factors at health centre and client level associated with variation in STI testing and counselling; quantify the variation attributable to health centre and client characteristics; and determine if provision of sexual health prevention/screening services increased with participation in CQI.

## Methods

### Setting

One hundred and thirty seven participating primary health centres in the ABCD NRP provided preventive health audit data between 2005 and 2014. These centres predominantly serve Aboriginal clients and include urban, regional and remote government- and community-controlled primary health care centres from 5 jurisdictions in Australia: NSW, NT, WA, SA and QLD. Based on a comprehensive care model and providing clinical services, health promotion and community care, Aboriginal PHC centres range in size and staffing and employ a range of health workers including general practitioners, nurses, allied health practitioners, mental health professionals, and Aboriginal health workers [12]. Some remote centres are staffed by a single nurse and Aboriginal Health Worker (AHW), with minimal administrative support and a visiting doctor while larger centres have several nurses, AHWs and doctors, and on-site allied health and specialised services.

### Data sources/collection

Details of the audit process have been documented elsewhere [13, 14]. In summary, clinical audits were conducted on a random sample of eligible client medical records at each health centre. For health centres with 30 or more eligible clients, the One21seventy training manual [15] provides guidance to auditors on how to draw random samples of medical records from eligible clients based on a precision of 90% or 95% confidence of that sample representing the population from which it was drawn. If the health centre’s population sample was less than 30 clients, the One21seventy training manual recommended that all records were audited [15]. Audits are conducted by health centre staff trained in the use of audit tools, supported by local or regional CQI facilitators. The preventive services audit tool, introduced in 2005, assesses the delivery of preventive health care against evidence-based guidelines for 15–54 year old clients without a history of chronic disease. The tool audits documentation of risk factors and brief interventions, scheduled services including sexual, oral, ear and eye health checks, and emotional wellbeing assessments. For inclusion in the preventive audit the client has to be 1) aged between 15 and 54 years; 2) have been a resident in the community for at least six of the last twelve months; 3) not have a diagnosis of chronic disease (diabetes, hypertension, coronary heart disease, chronic heart failure, rheumatic heart disease or chronic kidney disease); and 4) not be pregnant or less than 6 weeks postpartum at time of the audit. For the preventive tool, a service is considered as delivered if there is a clear record of delivery at least once in the last 24 months. Samples were stratified by age and gender. Analysis was confined to data related to sexual health care processes included within the clinical audit tool: documentation of a sexual and reproductive health (SRH) discussion, nucleic acid amplification test (NAAT) test for gonorrhoea and chlamydia and syphilis serology test. Other client information collected included age, gender, Aboriginal status, whether or not the client had attended in the previous 6 months, whether they had received an adult health check (AHC), reason for last attendance, which cadre of health provider they had seen first (doctor, nurse or AHW) and, for females, whether they had received a Pap smear. Health centre characteristics data collected included the location (urban, regional or remote), governance structure (government- or community-controlled), accreditation status, health service population, and CQI experience (number of clinical audit cycles conducted).

### Statistical analysis

STATA version 14 software (StataCorp, College Station, TX, USA) was used for statistical analysis. To assess delivery of STI testing and counselling, a sexual health delivery composite outcome measure was created which included sexual and reproductive health (SRH) discussion, NAAT test for gonorrhoea and chlamydia, and syphilis serology test by calculating the proportion of screening/counselling services received out of the total number of services the client should have received based on the audit protocol. The composite measure is expressed as a percentage; for instance if a client received all three services, this is expressed as “100% of recommended services were delivered to the client”. To determine variation in STI testing and counselling at the health centre level, the mean number of recommended tests/counselling delivered by each health centre to their eligible clients within an audit cycle or year was calculated. For logistic regression analysis, each composite measure score was converted to a binary outcome variable that categorised ‘higher’ performance as attaining 100% of service delivery, and anything less as ‘lower’ performance.

For unadjusted analysis, crude odds ratios were calculated to determine the association between the predictor variables (health centre and client factors) and the composite measure; the odds in this instance are ‘the odds of a patient receiving 100% of service delivery’ in comparison to the reference group. For multilevel logistic regression analysis a step-wise model was used starting with Model A to test the influence of health centres and jurisdictions on sexual health service delivery over audit years; health centre characteristics were added in Model B and in Model C, patient characteristics were included. This model allowed for the hierarchical structure of the data, that is, patients nested within health centres, nested within jurisdictions [14]. Only significant variables from the unadjusted regressions were included in models B and C. A sub-analysis was also conducted to determine factors associated with STI testing and counselling among female clients.

A median odds ratio (MOR) was used to quantify the variance in odds ratio scale, i.e. the increase in median probability of better delivery if a client moved from one randomly selected health centre/jurisdiction to another [16]. The MOR represents the median increased odds of a patient receiving ‘100% of service delivery’ if they were to change health centre/state. If the MOR = 1, there is no difference between groups. In addition, the proportional change in variance (PCV) was used to estimate the reduction in variance due to the step-wise introduction of variables into the model [17]. The significance level was set at p < 0.005 to account for the multiple associations tested between the outcome indicators and patient and health centre characteristics (Bonferroni correction [18]).

## Results

Of the 137 centres that audited for preventive services between 2005 and 2014, 45% were in the NT and 36% in QLD (Table 2). Seventy nine percent of the centres were located in remote areas; most (73%) were government-administered clinics; 49% had a service population equal or less than 500 people and 44% of health centres had completed 3 or more preventive audit cycles. Thirty one percent of the centres were accredited for the whole time period.

**Table 2:** Health centre characteristics by jurisdiction (Number and % of total)

		Far West NSW	Northern Territory	Queensland	South Australia	Western Australia	Total
Health centre level	Number of health centres audited	6	62	49	8	12	137
Location	Urban	0 (0)	1 (2)	3 (6)	4 (50)	3 (25)	11 (8)
	Regional	3 (50)	3 (5)	6 (12)	2 (25)	4 (33)	18 (13)
	Remote	3 (50)	58 (94)	40 (82)	2 (25)	5 (42)	108 (79)
Governance	Community controlled	6 (100)	19 (31)	1 (2)	4 (50)	7 (58)	37 (27)
	Government	0 (0)	43 (69)	48 (98)	4 (50)	5 (42)	100 (73)
Length of Accreditation^a^	Never accredited	4 (67)	34 (55)	5 (10)	2 (25)	0 (0)	45 (33)
	Accredited for some of the time	1 (17)	6 (10)	21 (43)	2 (25)	0 (0)	30 (22)
	Accredited for all of the time	1 (17)	13 (21)	22 (45)	4 (50)	3 (25)	43 (31)
	Missing	0 (0)	9 (15)	1 (2)	0 (0)	9 (75)	19 (14)
Service population	= < 500	2 (33)	35 (56)	26 (53)	3 (38)	1 (8)	67 (49)
	501-999	1 (17)	11 (18)	9 (18)	3 (38)	0 (0)	24 (18)
	= > 1000	3 (50)	16 (26)	14 (29)	2 (25)	11 (92)	46 (34)
CQI experience	Baseline audit only	0 (0)	15 (24)	7 (14)	4 (50)	2 (17)	28 (20)
	Completed 1–2 cycles	0 (0)	19 (31)	19 (39)	4 (50)	7 (58)	49 (36)
	Completed = > 3 cycles	6 (100)	28 (45)	23 (47)	0 (0)	3 (25)	60 (44)

^a^accreditation with Australian General Practice Accreditation Limited (AGPAL), Quality Improvement Council (QIC) or other, *CQI* continuous quality improvement, *NSW* New South Wales

There were 16,086 clinical records audited between 2005 and 2014, 41% in QLD and 39% in NT (Table 3); 51% of clients were aged 15–29 years (mean 31 years); 50% were males; 88% were Aboriginal. Sixty-eight percent of clients had attended the clinics in the last 6 months, 49% for acute care and only 8% specifically for sexual health; 50% were first seen by a nurse, 20% by an AHW. Around one-quarter (26%) of clients had received an AHC in the previous 24 months, 8% in NSW, 18% in QLD, 36% in the NT and 55% in SA.

**Table 3:** Client characteristics by jurisdiction (Number and % of total)

		Far West NSW	Northern Territory	Queensland	South Australia	Western Australia	Total
Client level	Number of client records audited	1293	6328	6639	675	1151	16086
Age group (years)	15-19	222 (17)	1,070 (17)	1,330 (20)	171 (25)	186 (16)	2,979 (19)
	20-24	204 (16)	1,305 (21)	1,143 (17)	89 (13)	202 (18)	2,943 (18)
	25-29	156 (12)	1,020 (16)	885 (13)	87 (13)	143 (12)	2,291 (14)
	30-34	127 (10)	784 (12)	756 (11)	81 (12)	126 (11)	1,874 (12)
	35-39	145 (11)	611 (10)	720 (11)	75 (11)	137 (12)	1,688 (10)
	40-44	193 (15)	694 (11)	773 (12)	59 (9)	159 (14)	1,878 (12)
	45-49	137 (11)	538 (9)	597 (9)	64 (9)	127 (11)	1,463 (9)
	50-54	109 (8)	306 (5)	435 (7)	49 (7)	71 (6)	970 (6)
Gender	Male	654 (51)	3163 (50)	3347 (50)	362 (54)	565 (49)	8091 (50)
	Female	639 (49)	3165 (50)	3292 (50)	313 (46)	586 (51)	7995 (50)
Aboriginal status	Aboriginal	983 (76)	6099 (96)	5451 (82)	657 (97)	1031 (90)	14221 (88)
	Non-Aboriginal	245 (19)	173 (3)	581 (9)	18 (3)	84 (7)	1101 (7)
	Not recorded	65 (5)	56 (1)	607 (9)	0 (0)	36 (3)	764 (5)
Time since last attendance	<6 months	732 (57)	4832 (76)	4419 (67)	383 (57)	594 (52)	10960 (68)
	>6 months	561 (43)	1496 (24)	2220 (33)	292 (43)	557 (48)	5126 (32)
Reason for last attendance	Well person’s check	62 (5)	758 (12)	670 (10)	211 (31)	76 (7)	1777 (11)
	Acute care	549 (42)	3237 (51)	3392 (51)	160 (24)	550 (48)	7888 (49)
	Sexual health	69 (5)	433 (7)	661 (10)	41 (6)	42 (4)	1246 (8)
	Other^a^	536 (41)	1773 (28)	1611 (24)	232 (34)	459 (40)	4611 (29)
	Did not attend in last 2 years/Not recorded	77 (6)	127 (2)	305 (5)	31 (5)	24 (2)	564 (4)
First seen health provider	AHW	119 (9)	1046 (17)	1302 (20)	318 (47)	403 (35)	3188 (20)
	Nurse	453 (35)	3834 (61)	3244 (49)	240 (36)	207 (18)	7978 (50)
	Doctors	419 (32)	875 (14)	1269 (19)	43 (6)	421 (37)	3027 (19)
	Others^b^	117 (9)	261 (4)	156 (2)	42 (6)	82 (7)	658 (4)
	Did not attend in last 2 years/Not recorded	185 (14)	312 (5)	668 (10)	32 (5)	38 (3)	1235 (8)
Adult health check/alternative health check	No	565 (44)	2670 (42)	5044 (76)	273 (40)	412 (36)	8964 (56)
	Yes	98 (8)	2301 (36)	1164 (18)	371 (55)	185 (16)	4119 (26)
	Did not attend in last 2 years/Not recorded	630 (49)	1357 (21)	431 (6)	31 (5)	554 (48)	3003 (19)

^a^mental health, immunisation, antenatal, other; ^b^Specialist, allied health, other; *AHW* Aboriginal Health Worker, *NSW* New South Wales

Of those who attended the centres in the 24 months prior to auditing (*n* = 15,212), 46% had a documented history of a sexual and reproductive health discussion, with the highest in NT (49%) (Table 4); 49% had a NAAT test for gonorrhoea and chlamydia documented (61% in NT and 49% in QLD); and 41% had a syphilis test, highest in NT (51%) and QLD (44%). For clients aged 15–29 years (data not shown), 52% had a documented history of a sexual health discussion, highest in SA (55%) and 56% had a documentation of a NAAT test for gonorrhoea and chlamydia (65% in NT and 59% in QLD). Forty seven percent of 15–29 years olds had a syphilis test with the highest in NT (55%) and QLD (53%).

**Table 4:** Percentage of clients (15–54 years) who received STI tests and counselling in previous 24 months

	Far West NSW	Northern Territory	Queensland	South Australia	Western Australia	Total
SRH discussion	26	49	46	45.5	31	46
NAAT for gonorrhoea and chlamydia test	17	61	49	27	23	49
Syphilis serology	7	51	44	13	14	41
Received all STI tests and SRH discussion^a^	6	41	33	8	12	31

^a^pre-2010 sexual health discussion data was not collected﻿; *NAAT* nucleic acid amplification test, *NSW* New South Wales, *SRH* Sexual and Reproductive Health

**Fig. 1:**
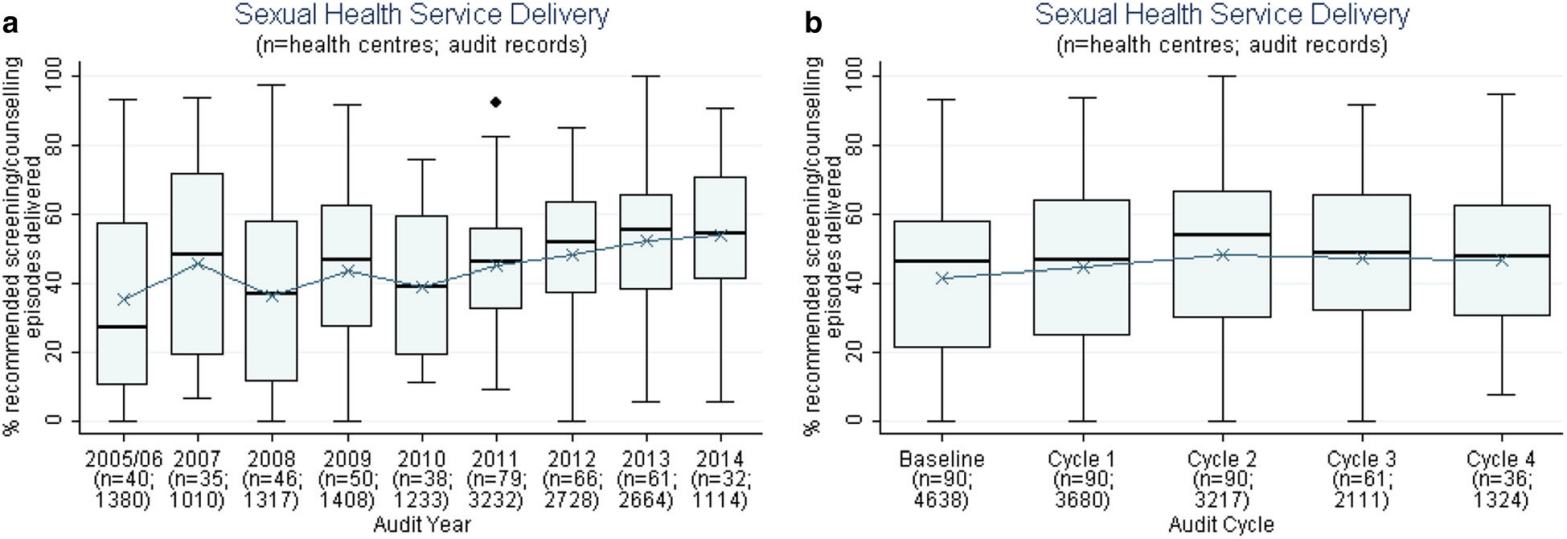
Sexual health related service delivery over time: Mean percentage of STI testing and counselling^a^ delivered per health centre over audit year (**a**) and cycle (**b**) (*n* = number of health centres; number of client records). ^a^Composite indicator includes NAAT for gonorrhoea and chlamydia, syphilis screen and reproductive health discussion

There was wide variation in delivery of STI tests and counselling between health centres (Fig. 1), ranging from 0% to 100% of services provided in some years/cycles. Between 2005/6 and 2014, there was an increase in the median percentage of STI tests and counselling services provided to clients (27% to 54%) and a reduction in the interquartile range (47.3% to 29.7%) suggesting a general trend in improvement in service delivery (Fig. 1a). For the 90 centres that conducted 3 or more CQI cycles, there were marginal increases in the median percentage of STI tests and counselling services over the 3 CQI cycles (46.1% to 53.8%) (Fig. 1b).

Unadjusted regression analysis of the sexual health delivery composite revealed a significant association with duration of participation in the ABCD CQI program, provision of an AHC, location, gender, age and Aboriginal status (Table 5). Health centres that provided AHCs were more likely to provide STI testing and counselling than those that had not provided AHCs; regional centres were less likely to provide these services compared to urban and remote health centres. Health centres that had conducted 1–2 CQI cycles were more likely to provide STI testing and counselling than those that had conducted a baseline audit, however, this was not sustained in subsequent cycles. Those clients who were female, aged 15–39 years, and Aboriginal were more likely to receive STI testing/counselling than those who were male, aged older than 39 years and non-Aboriginal, respectively.

**Table 5:** Unadjusted and adjusted multilevel logistic regression analysis of health centre and client level factors on composite indicator of recommended STI testing and counselling services (*n* = 137 health centres; 15,212 patient records)

Outcome is 100% service delivery		Unadjusted		Model A		Model B		Model C	
Predictors	Fixed effects	OR	95% CI	OR	95% CI	OR	95% CI	OR	95% CI
Audit Year	2007			1.00	(reference)	1.00	(reference)	1.00	(reference)
	2008			0.65	(0.46-0.91)	0.79	(0.55-1.12)	0.82	(0.57-1.19)
	2009			0.65	(0.47-0.92)	0.73	(0.51-1.04)	0.74	(0.51-1.06)
	2010			0.44	(0.31-0.63)^b^	0.50	(0.34-0.73)^b^	0.49	(0.33-0.73)^b^
	2011			0.34	(0.24-0.47)^b^	0.43	(0.30-0.63)^b^	0.43	(0.29-0.63)^b^
	2012			0.51	(0.36-0.72)^b^	0.56	(0.38-0.83)^a^	0.57	(0.38-0.85)
	2013			0.61	(0.43-0.86)	0.71	(0.48-1.05)	0.71	(0.48-1.06)
	2014			0.57	(0.40-0.82)^a^	0.66	(0.43-1.00)	0.62	(0.40-0.94)
*Health Centre Characteristics*									
Location	Urban	1.00	(reference)			1.00	(reference)	1.00	(reference)
	Regional	0.25	(0.12-0.56)^a^			0.41	(0.19-0.86)	0.38	(0.18-0.80)
	Remote	1.71	(0.88-3.34)			2.09	(1.12-3.90)	1.98	(1.05-3.74)
Governance	Community-controlled	1.00	(reference)						
	Government operated	0.78	(0.49-1.23)						
Service population	≥1000	1.00	(reference)						
	>500- < 1000	1.30	(0.78-2.17)						
	≤500	1.31	(0.88-1.96)						
Length of accreditation^a^	Never accredited	1.00	(reference)						
	Accredited for some of the time	1.45	(0.94-2.24)						
	Accredited for all of the time	0.73	(0.49-1.06)						
Duration of participation in ABCD CQI project	Baseline	1.00	(reference)			1.00	(reference)	1.00	(reference)
	1-2 cycles	1.49	(1.35-1.64)^b^			1.30	(1.12-1.50)^b^	1.34	(1.16-1.55)^b^
	≥3 cycles	1.11	(0.98-1.24)			0.97	(0.78-1.20)	1.01	(0.81-1.26)
Provided adult health check^b^	No	1.00	(reference)			1.00	(reference)	1.00	(reference)
	Yes	3.17	(2.88-3.50)^b^			3.25	(2.94-3.59)^b^	3.40	(3.07-3.77)^b^
*Patient Characteristics*									
Sex	Male	1.00	(reference)					1.00	(reference)
	Female	1.41	(1.30-1.52)^b^					1.45	(1.33-1.57)^b^
Age (years)	15-19	1.82	(1.48-2.22)^b^					2.10	(1.67-2.63)^b^
	20-24	3.21	(2.63-3.92)^b^					3.84	(3.07-4.80)^b^
	25-29	2.82	(2.30-3.47)^b^					3.01	(2.39-3.79)^b^
	30-24	2.15	(1.74-2.65)^b^					2.35	(1.85-2.97)^b^
	35-39	1.72	(1.38-2.13)^b^					1.77	(1.39-2.26)^b^
	40-44	1.35	(1.08-1.67)					1.36	(1.07-1.73)
	45-49	1.17	(0.93-1.47)					1.27	(0.99-1.63)
	50-54	1.00	(reference)					1.00	(reference)
Aboriginal Status	Non-Aboriginal	1.00	(reference)					1.00	(reference)
	Aboriginal	2.10	(1.70-2.61)^b^					1.46	(1.15-1.84)^a^
	Not recorded	1.55	(1.13-2.12)					1.09	(0.77-1.55)
Random effects (intercepts)									
State (variance (SE))				1.05	(0.71)	0.54	(0.39)	0.55	(0.395)
MOR_STATE_				2.66		2.02		2.03	
*PCV (% explained variance)*						*48%*		*47%*	
Health Centre (variance (SE))				0.84	(0.14)	0.54	(0.09)	0.55	(0.095)
MOR_HC_				2.39		2.01		2.03	
*PCV (% explained variance)*						*36%*		*34%*	
State & Health Centre (variance)				1.89		1.08		1.11	
MOR_STATE-HC_				3.70		2.69		2.73	
*PCV (% explained variance)*						*43%*		*41%*	
Patient (variance (SE))				0.28	(0.14)	0.12	(0.055)	0.03	(0.015)
*PCV (% explained variance)*						*59%*		*89%*	

*CQI* continuous quality improvement, *MOR* Median Odds Ratio: odds of receiving above median service delivery if client was to change health centre or jurisdiction, *OR* Odds Ratio, *PCV* proportional change in variance: per cent variation explained in odds for better healthcare delivery by introduction of health centre or client level factors, *SRH* Sexual and Reproductive Health

Due to multiple testing, the significance level is set at *p* < 0.005 (Bonferroni correction); ^a^ *p* < 0.005; ^b^ *p* < 0.0001

^a^(*n* = 13,989 with accreditation status recorded); ^b^(*n* = 13,235 with AHC recorded, introduced to audit tool in 2007)

In the adjusted analysis of the sexual health delivery composite, health centre factors that remained associated with higher levels of STI testing and counselling were provision of an AHC (adjusted odds ratio (AOR) 3.40; 95% confidence interval (CI) 3.07-3.77) and having conducted 1–2 cycles of CQI (AOR 1.34; 95% CI 1.16-1.55) (Table 5: Model C). Client level factors significantly associated with higher levels of sexual health care delivery were being female (AOR 1.45; 95% CI 1.33-1.57), and Aboriginal compared to non-Aboriginal (AOR 1.46; 95% CI 1.15-1.84) with those aged 20–24 years and 25–29 years having the highest likelihood of receiving STI testing and counselling (AOR 3.84; 95% CI 3.07-4.80; AOR 3.01; 95% CI 2.39-3.79 respectively) compared to those aged 15–19 years and over 29 years. In the sub-analysis for females, Pap smear was significantly associated with STI testing and counselling in unadjusted and adjusted analysis with those who had had a Pap smear significantly more likely to receive STI testing and counselling (AOR 4.39; 95% CI 3.84-5.03; *p* < .0001) (Table 6).

**Table 6:** Adjusted multilevel logistic regression analysis of health centre and client level factors on composite indicator of recommended STI testing and counselling services for female clients (*n* = 137 health centres; 7,670 female patient records)

Outcome is 100% service delivery		Model A		Model B		Model C	
Predictors	Fixed effects	OR	95% CI	OR	95% CI	OR	95% CI
Audit Year	2007	1.00	(reference)	1.00	(reference)	1.00	(reference)
	2008	0.73	(0.45-1.18)	1.04	(0.63-1.74)	1.13	(0.66-1.91)
	2009	0.70	(0.43-1.14)	0.87	(0.52-1.45)	0.88	(0.52-1.50)
	2010	0.58	(0.35-0.95)	0.75	(0.44-1.29)	0.75	(0.43-1.30)
	2011	0.44	(0.27-0.71)^a^	0.61	(0.36-1.03)	0.61	(0.36-1.05)
	2012	0.61	(0.38-1.00)	0.69	(0.41-1.17)	0.70	(0.41-1.21)
	2013	0.74	(0.45-1.20)	0.83	(0.49-1.42)	0.83	(0.48-1.45)
	2014	0.51	(0.31-0.85)	0.55	(0.31-0.96)	0.49	(0.27-0.87)
*Health Centre Characteristics*							
Location	Urban			1.00	(reference)	1.00	(reference)
	Regional			0.34	(0.16-0.72)	0.34	(0.16-0.73)
	Remote			1.05	(0.56-1.98)	1.10	(0.59-2.06)
Duration of participation in ABCD CQI project	Baseline			1.00	(reference)	1.00	(reference)
	1-2 cycles			1.41	(1.16-1.70)^b^	1.47	(1.21-1.79)^b^
	≥3 cycles			1.28	(0.99-1.67)	1.38	(1.05-1.80)
Provided adult health check^a^	No			1.00	(reference)	1.00	(reference)
	Yes			2.11	(1.83-2.44)^b^	2.24	(1.93-2.60)^b^
Provided Pap smear	No			1.00	(reference)	1.00	(reference)
	Yes			3.66	(3.24-4.14)^b^	4.39	(3.84-5.03)^b^
*Patient Characteristics*							
Age (years)	15-19					4.53	(3.23-6.34)^b^
	20-24					5.62	(4.04-7.81)^b^
	25-29					3.82	(2.73-5.34)^b^
	30-24					3.21	(2.28-4.51)^b^
	35-39					2.18	(1.53-3.10)^b^
	40-44					1.54	(1.08-2.19)
	45-49					1.41	(0.97-2.03)
	50-54					1.00	(reference)
Aboriginal Status	Non-Aboriginal					1.00	(reference)
	Aboriginal					1.33	(0.98-1.81)
	Not recorded					0.96	(0.60-1.54)
Random effects (intercepts)							
State (variance (SE))		0.91	(0.63)	0.55	(0.40)	0.56	(0.40)
Health Centre (variance (SE))		0.76	(0.14)	0.44	(0.087)	0.43	(0.085)
State & Health Centre (variance)		1.67		0.99		0.98	
Patient (variance (SE))		0.31	(0.16)	0.11	(0.057)	0.023	(0.013)

*CQI* continuous quality improvement, *MOR* Median Odds Ratio: odds of receiving above median service delivery if client was to change health centre or jurisdiction, *OR* odds Ratio, *PCV* proportional change in variance: per cent variation explained in odds for better healthcare delivery by introduction of health centre or client level factors, *SRH* Sexual and Reproductive Health

Due to multiple testing, the significance level is set at *p* < 0.005 (Bonferroni correction); ^a^ *p* < 0.005; ^b^ *p* < 0.0001

^a^(*n* = 6,645 with AHC recorded, introduced to audit tool in 2007)

The variation in delivery of the sexual health composite was reduced between jurisdictions and health centres after accounting for health centre factors as shown by the reduction in the MOR_STATE-HC_ from 3.70 (Table 5; Model A) to 2.69 (Table 5, Model B). The PCV for STI testing and counselling in Model B (Table 5) showed that the addition of health centre factors explained 43% of variation across jurisdictions and health centres. The addition of client factors in the model had little impact on explaining the remaining variation.

## Discussion

This study is the first of its kind in Australia, providing STI testing and counselling data from the largest and longest running CQI program within Aboriginal primary care, across five states and territories, involving government- and community-controlled, urban, regional and remote health centres. The study quantified the extent of variation in STI testing and counselling within Aboriginal primary health care centres and demonstrated that a significant proportion of variation was explained by health centre factors. It has shown that a number of jurisdictions and Aboriginal PHC centres are achieving high rates of STI testing but that there are substantial opportunities for improvement in the STI testing and counselling in many Aboriginal PHC centres.

Wide variation in service delivery in Aboriginal PHC centres has been documented in previous studies of CQI in diabetes care [14], preventive care [19] and cardiovascular care [20]. In a study of CQI in diabetes care, variation reduced over audit cycles with lower performing centres showing improvements in adherence to best practice, and with a significant association between duration of CQI participation and the improvement in diabetes care processes [14]. In contrast, though there were marginal improvements in the median percentage of STI tests and counselling services delivered over CQI cycles, the current analysis shows no clear association between CQI experience and higher levels of documented delivery of sexual health care, with continuing low levels of documented STI testing and counselling in some health centres despite participation in several cycles of CQI. The preventive tool audit process was not focused on sexual health delivery but on preventive care in general, with health centres setting goals and implementation strategies based on audit findings, context-specific priorities and available resources. Following on from the findings of Matthews et al. [14], analysis of the diabetes data that shows there was an association of improved level of care with longer duration of participation in CQI, the findings from this analysis of the sexual health data suggest that without specific focus on sexual health, a more general preventive health CQI process may not necessarily result in improved sexual health care in the medium to long term, even though there is an association between duration of CQI in preventive health and STI counselling and testing over the initial 1–2 years. Further understanding about whether it was a lack of prioritisation of sexual health or other factors that impacted on delivery of sexual health care will have implications for the development of tailored strategies to improve the delivery of sexual health care in this context. As pointed out in Larkins et al. 2016 [21], trends in quality of care varied widely between health services across different audit tools, “point[ing] to the need for a deeper or more nuanced understanding of factors that moderate the effect of CQI on health service performance”.

This study also found a positive association between Pap smear and STI testing in female clients. Cervical screening among the Aboriginal female population is important given that the incidence of and mortality from cervical cancer is higher among Aboriginal women compared to non-Aboriginal women [22, 23] but also because a Pap smear provides an opportunity for STI testing [24]. Opportunities to maintain and intensify cervical screening rates among Aboriginal women is key to reducing cervical cancer incidence and mortality rates but also for maintaining STI testing rates among Aboriginal women [24]. Changes will occur in Australia’s cervical cancer screening program from 2017 which will incorporate a primary Human Papillomavirus (HPV) test every 5 years and reduce the requirement for 2-yearly Pap tests as occurs currently [25] and could impact on STI testing rates among female general practice clients. Data collection as occurs through ABCD NRP/One21seventy provide important infrastructure by which to measure the impact of such policy changes on a key target group.

Our findings that females and clients aged 20–24 years receive higher levels of STI testing in comparison to males and other age groups, respectively, has been documented elsewhere [26]. Kong et al. [5] found that the rate of STI testing at general practice was higher in females than males (12.5 vs. 3.7%) and in 20–24 year olds than 16–19 and 25–29 year age groups (9.0%, 8.7% and 6.6% respectively) with the highest testing rates among 20–24 year olds in the NT (21.9%) and females in the NT (31.2%). More women are screened for STIs in conjunction with a Pap smear and following discussions about contraception. However, it is also likely to reflect different health seeking behaviour [27] and their higher access, compared to males, to more culturally appropriate primary care given the largely female health workforce in regional and remote Australia [28]. A focus on opportunities to increase the number of males being screened for STIs at primary health care is essential in order to reduce the overall burden of infections. However, this necessitates greater understanding of the specific barriers to access of care for males, which have included prioritising time for health care, lack of male service providers and poorer access to health care [27]. Similarly, interventions to increase STI testing rates among 15–19 year olds should be prioritised considering the high incidence rates in this age group [29].

Among health centres that participated in the ABCD CQI program, the NT and QLD health centres were the highest performing, with the highest documented testing rates for gonorrhoea, chlamydia and syphilis compared to other jurisdictions. Known facilitators of best practice in sexual health care include motivated and knowledgeable health workers, creativity and innovation in delivery of services including outreach services, offering STI screening as part of the AHC to reduce the stigma associated with STI testing, use of nurses and other practice staff to test clients, and provision of incentives [30, 31]. Barriers to best practice include lack of knowledge and time, workload load pressures, competing priorities and gender-related, cultural, structural and organisational factors [30, 31], with major concerns around privacy and confidentiality particularly in smaller, more remote Aboriginal communities [32, 33]. In both the NT and QLD there was significant investment in CQI infrastructure between 2009 and 2013, including the establishment of regional CQI facilitator networks and state-wide CQI planning and governance committees [34, 35], which could explain the higher levels of service delivery in these jurisdictions. It is noteworthy that an ongoing syphilis outbreak in northern Australia which began around January 2011, has resulted in a renewed effort in monitoring, surveillance and management of STIs in the region [36] and may have impacted on the STI testing rates in these jurisdictions. An additional explanatory factor for the higher STI testing rates could be the concurrent STI-specific CQI intervention, the STRIVE program, a large randomised controlled trial which was conducted among 68 remote Aboriginal communities in the NT, QLD and services in northern WA between 2009 and 2014 [8]. Previous studies have shown that STI-specific interventions have a positive impact on STI testing rates [7], however, delineation of the impact of these factors on delivery of sexual health services in the NT and QLD will provide a better understanding around what is needed for better delivery of care. Transferrable strategies that can be used to improve service delivery in other regions and within health centres with relatively lower documented evidence of adherence to best practice guidelines are needed. Improving delivery of care among services at the lower end of the range of service delivery is critically important to reducing inequities in care and raising the standard of care at the population level.

Clients had three times the odds of receiving testing for all three STIs and counselling if they had an AHC. Health checks are primarily diagnostic and preventive, and the Medicare Benefits Scheme (MBS) allows for the Health Assessment for Aboriginal and Torres Strait Islander People Item 715 (MBS 715) at least once every nine months including a sexual health history, Pap smears and testing for STIs [37]. Combining STI testing with an AHC may be a way of normalising STI screening and reducing stigma by removing emphasis from the individual’s risk behaviour [30]. Our analysis shows that only a quarter of clients had received an AHC within 24 months and an increase in AHCs would, by extension, lead to an increase in STI testing. However, yearly AHCs may not be sufficient to control the high rates of STI transmission, particularly in the 15–19 year old age group that has very high documented incidence of chlamydia and gonorrhoea [29]. Adult health checks and Pap smears could therefore be useful, in conjunction with other measures, in improving STI testing in health centres and among client groups with low testing rates where STI-related stigma is an issue. As our study illustrated, the majority of the clients (92%) attended the health centres for reasons other than a sexual health assessment, so AHCs provide an opportunity for screening and reducing the burden of disease in otherwise ‘well adults’. Considering that up to 80% of chlamydia infections are asymptomatic [38] as well as a significant proportion of gonorrhoea infections [39], clients attending the health centre for reasons other than a sexual health consult could benefit from STI screening. There are a range of patient-, interpersonal-, health service-, community- and policy-level barriers to follow-up care following a health assessment, including high mobility, costs, lack of transport, negative past experiences, poor communication and lack of emphasis on follow up at health system and policy levels [40]. Strategies at various levels of the system to address barriers to follow-up following STI testing are necessary to ensure continued benefits considering the substantial investment in health assessments in Australia [40].

There are several limitations to this study that have to be taken into consideration when interpreting the findings. The auditing process collects a limited amount of data so potential important factors may not be measured; health centre factors, contextual factors in the wider community and structural and legislative factors that may impact on delivery of care are not readily measured and quantified. Workload pressures at health centre level were considered during development of the audit tools; selecting indicators of relevance to Aboriginal primary care centres reflects the service-orientation of the ABCD projects. Lack of workforce data both in terms of number and cadre of health workers at service level and data on access to male and female service providers precludes analysis through our current dataset of the impact of these factors on sexual health service delivery in this context. The fact that less than 50% of variation could be accounted for by the available health centre and client factors indicates that there are other factors underlying variation. Furthermore, the variation in documented STI counselling and testing between regions reflect a differing background STI prevalence, local and regional guidelines and clinical practice. Variation in testing may also be a reflection of access to laboratory services, particularly in rural and remote regions. It is expected that point-of-care testing will enhance early diagnosis of STIs, particularly in remote areas where there are significant delays in the management of STIs [41]. Future studies, including qualitative assessments, could elucidate the impact of these and other contextual factors within health services with higher levels of performance in order to understand the less tangible factors associated with higher quality of sexual health care.

Another limitation of this study is that auditing depends on documentation of care in the client’s medical record and audit data may not be a true reflection of actual service delivery. This study could have underestimated the number of STI tests and sexual health consultations provided by the health centre, for instance in circumstances where health centres facilitate community outreach to increase clients’ access to STI testing and counselling if this is not incorporated back into patient information record systems. It is also not possible to estimate the number of clients who could have accessed these services outside their regular health centres, for instance, highly mobile persons or those who have access to other health services e.g. mainstream general practices. The preventive audit is limited to 15–54 year olds and excludes pregnant and postpartum women and those with a diagnosis of a chronic disease; this may lead to an underestimation of STI testing and counselling conducted by health centres. Underlying assumptions in the analysis were that all clients were sexually active and that all females had an intact cervix, that is, not undergone a hysterectomy, thus possibly overestimating the need for STI testing and counselling. Participation in One21seventy and the ABCD National Research Partnership is voluntary; health centres were invited to participate in the ABCD/One21seventy program [9] and furthermore, asked to provide their data to the ABCD NRP for research purposes [10], of which approximately two-thirds did. Therefore data are not representative of all primary health centres, regionally or nationally, and largely reflect service activity in remote health centres in the NT and QLD.

This study has elucidated several areas for improvement and demonstrated that variation in STI-related service delivery between health services is highly dependent on health centre factors rather than client factors, which highlights the critical need for structural and organisational changes, rather than client-centred interventions, to overcome the specific barriers to provision of adequate care and improve sexual health care delivery. Organisational-level interventions, including creation of multidisciplinary clinical teams, revision of professional roles, skills mix changes, and structural changes [42], could help increase the number and cadre of health professionals involved in sexual health care and overcome specific barriers to provision of adequate levels of care. The analysis has established a significant association between AHCs and Pap smears with higher levels of STI testing and counselling, suggesting that AHCs and Pap smears act as catalysts for STI testing, particularly in health centres with lower levels of documented care and in client groups with low STI testing rates. Ultimately the primary health workforce and health system will need to develop innovative ways to increase STI testing to help reduce the burden of STIs in the Aboriginal population as a means to reduce variation and enhance equity in quality of sexual health care.

## Conclusions

This study has shown that a number of jurisdictions and Aboriginal PHC centres are achieving high rates of STI testing, providing an opportunity for better understanding of what contributes to higher levels of performance in sexual health care. There are a significant number of health centres with relatively low documented evidence of adherence to best practice and analysis shows that variation is mainly attributed to health centre factors. STI-related service delivery could be substantially improved through focussed efforts to support delivery of services in health centres, and to groups of clients, with relatively lower documented evidence of adherence to best practice guidelines. Documentation of AHCs and Pap smear testing is associated with more testing for STIs and may contribute to earlier detection and treatment of STIs, and STI testing should be included with these interventions. Nevertheless, all PHC personnel should be encouraged to consider STI screening for all young men and women presenting to the health centre for any reason, at every opportunity, without necessarily waiting until the next AHC or Pap smear is due, in conjunction with other approaches to increase STI testing in high risk groups. Further analysis of local, regional and jurisdictional factors that impact on sexual health service delivery will assist health workers, PHC service managers and policy makers develop targeted interventions to improve service delivery. These approaches could help enhance equity in quality of sexual health care, contribute to the reduction in the high burden of STIs in the Aboriginal population in Australia and contribute to an overall improvement in quality of life for Aboriginal Australians.

## Abbreviations

ABCD: Audit and best practice for chronic disease
AGPAL: Australian general practice accreditation limited
AHC: Adult health check
AHREC: Aboriginal health research ethics committee
AHW: Aboriginal health worker
CARPA: Central Australian rural practitioners association
CQI: Continuous quality improvement
HC: Health centre
HREC: Human research ethics committee
MBS: Medicare benefits scheme
MOR: Median odds ratio
NAAT: Nucleic acid amplification test
NACCHO: National Aboriginal community controlled health organisation
NHMRC: National health and medical research council
NRP: National research partnership
NSW: New South Wales
NT: Northern Territory
PCV: Proportional change in variance
PHC: Primary health care
QIC: Quality improvement council
QLD: Queensland
RACGP: Royal Australian college of general practitioners
SA: South Australia
SHIMMER: Sexual health quality improvement project in NSW
SRH: Sexual and reproductive health
STI: Sexually transmitted infection
STRIVE: STI in remote communities: Improved and enhanced primary health care
WA: Western Australia
